# Potential applications of insect symbionts in biotechnology

**DOI:** 10.1007/s00253-015-7186-9

**Published:** 2015-12-14

**Authors:** Aileen Berasategui, Shantanu Shukla, Hassan Salem, Martin Kaltenpoth

**Affiliations:** Insect Symbiosis Research Group, Max Planck Institute for Chemical Ecology, Jena, Germany; Biochemistry Department, Max Planck Institute for Chemical Ecology, Jena, Germany; Department of Entomology, Max Planck Institute for Chemical Ecology, Jena, Germany; Department for Evolutionary Ecology, Institute of Zoology, Johannes Gutenberg University, Mainz, Germany

**Keywords:** Symbiosis, Mutualism, Paratransgenesis, Incompatible insect technique, Biotechnology

## Abstract

Symbiotic interactions between insects and microorganisms are widespread in nature and are often the source of ecological innovations. In addition to supplementing their host with essential nutrients, microbial symbionts can produce enzymes that help degrade their food source as well as small molecules that defend against pathogens, parasites, and predators. As such, the study of insect ecology and symbiosis represents an important source of chemical compounds and enzymes with potential biotechnological value. In addition, the knowledge on insect symbiosis can provide novel avenues for the control of agricultural pest insects and vectors of human diseases, through targeted manipulation of the symbionts or the host-symbiont associations. Here, we discuss different insect-microbe interactions that can be exploited for insect pest and human disease control, as well as in human medicine and industrial processes. Our aim is to raise awareness that insect symbionts can be interesting sources of biotechnological applications and that knowledge on insect ecology can guide targeted efforts to discover microorganisms of applied value.

## Introduction

Insects engage in a remarkable array of symbiotic interactions with microorganisms, which range from parasitic to mutualistic relationships. Among mutualisms, most of the best described associations are based on nutritional or defensive services provided by the symbionts to their hosts. In defensive interactions, the microorganisms protect their host against pathogens, parasites, parasitoids, or predators, often through the production of antimicrobial compounds or toxins (Flórez et al. 2015), whereas in nutritional mutualisms, they provide nutrients such as amino acids and vitamins or digestive enzymes that aid in the degradation of fastidious dietary polymers or the detoxification of noxious secondary metabolites (Douglas 2009). Mutualistic relationships have played a major role in the evolution of insects, allowing them to exploit ecological niches that would have otherwise remained inaccessible (Sudakaran et al. 2015).

**Fig. 1:**
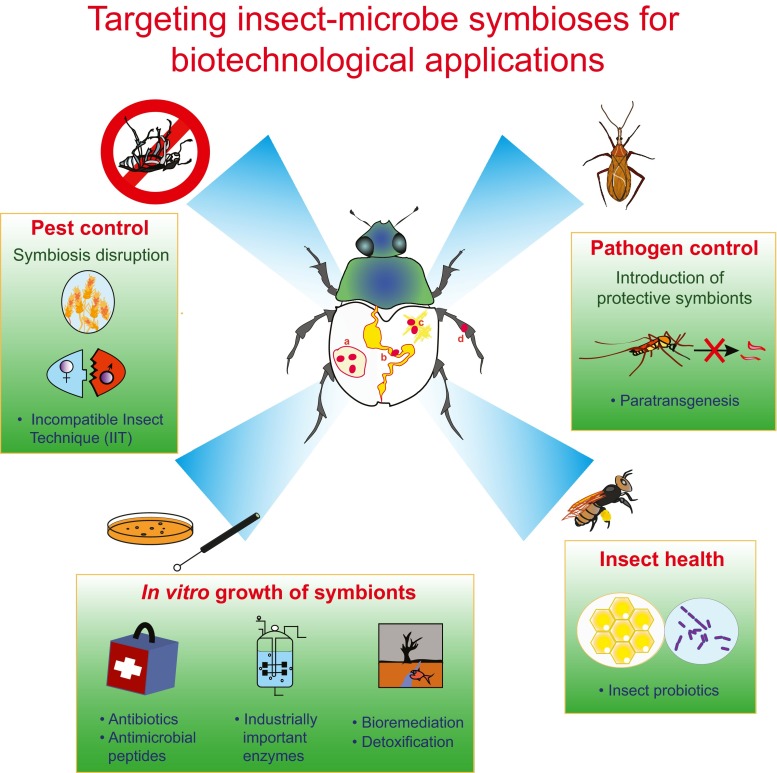
Biotechnological applications of targeting microbial symbionts in insects located (**a**) in specialized compartments (e.g., bacteriomes), (**b**) in the gut of the insect, (**c**) in insect tissues outside the gut (e.g., fat body), or (**d**) on the insect’s cuticle. Targeting these symbiotic interactions can have broad applications in controlling populations of insect pests, increasing the survival of beneficial insects, and utilizing the symbionts for industrially important processes

From a biotechnological perspective, symbiotic microorganisms constitute promising and mostly untapped sources for potential applications in medicine, bioremediation, industrial processes, and agriculture. As with free-living microbes, the efficiency of metabolites and enzymes produced by symbionts has been optimized for over millions of years by natural selection. In contrast to their free-living counterparts, however, symbiotic products have been tested for their efficacy in a eukaryotic host, increasing the chances of successful applications by humans due to the reduced risk of harmful side effects. In general, the knowledge on host-microbe interactions can be exploited in two different ways for biotechnological use (Fig. 1): (1) by targeting or utilizing symbiotic interactions to control agricultural pests or vector-borne diseases or to improve the health of economical important insects such as honeybees and (2) by the application of symbiont-produced compounds such as small bioactive molecules or enzymes for pharmaceutical use or industrial processes.

There is an accumulating body of research and review articles that have touched on insect symbiosis as biotechnological resources (Douglas 2007; Chaves et al. 2009; Jurkevitch 2011; Crotti et al. 2012; Ramadhar et al. 2014). However, we believe that the present minireview finds its value in providing a comprehensive overview of contexts in which insect symbiosis research may yield biotechnologically exploitable results, thereby bridging the areas of symbiosis research and biotechnology and raising awareness that the knowledge on insect ecology and symbiosis allows to target particular systems that are promising sources of biotechnologically interesting symbionts.

### Implications of insect symbiosis for biological control of agricultural pests

The obligate reliance of many insects on their microbial partners provides a potential target for the biological control of devastating agricultural pests. As such, numerous studies have examined the importance of the associated microorganisms to host fitness and feeding ecology in an effort to manipulate these partnerships and render insect pests more vulnerable to broad-scale measures of population control by targeting the bacterial symbionts.

Some of the best-studied animal-bacterial mutualisms feature insects specializing on economically important crops, including aphids, whiteflies, mealybugs, and stinkbugs, as well as many others. Numerous manipulations have been administered to hinder the development and survivorship of insect pests by targeting the bacterial partner, mainly through the application of antibiotics (Baumann 2005) and/or by disrupting the symbiont’s transmission route to the next host generation (Salem et al. 2015). While effective in highly controlled conditions (Nogge 1976; Tsuchida et al. 2004; Hosokawa et al. 2007; Salem et al. 2013), the use of these techniques to target pests in agricultural fields is either unfeasible technically, economically, and/or ethically, considering the drawbacks associated with antibiotic resistance as a by product of the wide-scale application of these compounds. However, based on the increasing interest in the development and use of antimicrobial peptides (AMPs) as a tool to control bacterial populations, coupled with the genetic tractability of some agricultural crops to produce AMPs (Francois et al. 2002), wide-scale delivery of these compounds via heterologous expression in the host plant may represent a targeted, cost-effective approach that may have broad implications, both in scale and implementation. It is important to note, however, that AMPs—like antibiotics—could potentially harm mutualistic bacteria of plants and beneficial insects, so the ecological implications of this approach must be carefully investigated.

Beyond demonstrations that the microbial associates of these agricultural pests influence survivorship and development, there is now growing evidence that the symbionts can also broaden the range of food plants that the insect host can utilize, which has profound implications for the economic risks that accompany a switch to an agricultural crop. While chromosomal loci of pea aphids (*Acyrthosiphon pisum*) have long been considered as predictors of host plant specialization, findings from Tsuchida and colleagues (2004) demonstrated that symbiotic bacteria can be similarly impactful, as evidenced by the ability of aphids to utilize white clover following specific infection by a facultative symbiont. Similarly, in whiteflies (*Bemisia tabaci*) specializing on sweet potatoes, a recent study has shown that infection by the endosymbiont *Rickettsia* results in insects that exhibit greater fecundity, faster development, higher survivorship, and increased production of females than observed in uninfected insects (Himler et al. 2011). Such benefits are thought to have contributed to the spread of the symbiont across whitefly populations at an unprecedented rate, thereby significantly impacting the ecology and invasive biology of its host. Along similar lines, the pest status of shieldbugs strongly correlates with the genotypic signature of their bacterial partner (Hosokawa et al. 2007). Here, the legume-feeding shieldbug *Megacopta cribraria* suffered low survivorship and reproductive success when provisioned with soybeans as a sole food source; such effects were reversed when the insect’s obligate symbionts were exchanged with symbiotic strains originating from the soybean-specializing shieldbug *Megacoptapunctatissima* (Hosokawa et al. 2007). Consistent with this finding, an invasive population of *M*. *cribraria* in North America, which is utilizing soybeans, has a symbiont population with an overall nucleotide and functional profile resembling that of the Asian pest-conferring symbionts in *M*. *punctatissima* (Brown et al. 2014).

Classical methods of biological control of agricultural pests take advantage of parasitoids to reduce the insect pest population. In this context, knowledge on insect symbiosis could be of applied value, as some *Wolbachia* strains harbored by parasitoid insects induce thelytokous parthenogenesis in their hosts (Arakaki et al. 2000). Since only female parasitoids kill their hosts, a parthenogenetic phenotype in a parasitoid would have several advantages on insect pest control over the sexual one as noted by Stouthamer (1993) and Bourtzis (2008): (i) a drop in the cost of mass-producing parasitoids for release due to the fact that no males are produced, (ii) rapid population growth due to the higher number of females, and (iii) easier establishment because no mating is required.

### Symbiosis as a tool to limit vector-borne diseases

The use of microbial symbionts to limit the prevalence and competence of insect vectors of human diseases has been heralded as a promising research area to control the incidence of numerous devastating diseases, including malaria, dengue, yellow fever, and Chagas. Currently, two of the most active research areas include (1) the genetic transformation of bacterial symbionts to express molecules targeting the disease agent in the insect vector (paratransgenesis) and (2) manipulating the vector through the utilization of microbes that shorten life span and lower fertility of the insect host or that reduce its susceptibility to pathogens or parasites.

For the former, the most extensively developed model involves targeting the insect vector (*Rhodnius prolixus*) of the Chagas-causing protozoan *Trypanosoma cruzi* through the manipulation of the insect gut flora (Ben Beard et al. 2002). Early application of paratransgenesis in this system focused on the ability to genetically transform the gut symbiont *Rhodococcus rhodnii*, which co-localizes in the midgut with *T*. *cruzi*, in order to produce anti-trypanosomal effector molecules that target the parasite in the insect’s gut (Durvasula et al. 1997). The transmission ecology of *R*. *rhodnii* (extracellular through coprophagy) and its amenability for in vitro cultivation and genetic transformation has presented the system as a useful platform to apply paratransgenesis as a way to limit the transmission of *T*. *cruzi*, as has been shown in semi-field trials (Durvasula et al. 1999). Additionally, Taracena and colleagues (2015) successfully introduced *Escherichia coli* expressing dsRNA for *Rhodnius* heme-binding protein and catalase into the gut of *R*. *prolixus*, which had serious fitness consequences for the bug by inducing systemic RNAi. Likewise, paratransgenesis has been used in tsetse flies to engineer *Sodalis glossinidius* to release anti-trypanosome nanobodies (antigen-binding molecules) in the host gut (De Vooght et al. 2014). A significant problem for paratransgenetic control of pest insects or disease vectors remains the delivery of manipulated bacteria to the insect under field conditions. However, an important step into this direction was the recent establishment of a targeted delivery system for genetically engineered bacteria using microencapsulation to control the spread of Pierce’s disease by glassy-winged sharpshooters (*Homalodisca vitripennis*) under simulated field conditions (Arora et al. 2015).

In an effort to inhibit the transmission of mosquito-borne filariasis, *Wolbachia* strain wMelPop was successfully used to provoke a sustained insect immune response that incurs a heavy metabolic burden on the insect host (Kambris et al. 2009). This results in a shorter life span of infected mosquitoes, which reduces the possibility of disease transmission, since the parasite requires a long incubation time relative to the average life span of an individual mosquito. The upregulation of the immune system was also found to eliminate lymphatic filariasis from the vector, thereby disrupting its transmission (Kambris et al. 2009). *Wolbachia* wMelPop has also been successfully utilized to reduce the life span of laboratory cultures of the mosquito vector of dengue (*Aedes aegypti*), thereby compromising the ability of the virus to establish in the insect and effectively blocking subsequent transmission to a mammalian host (McMeniman et al. 2009). Most promising, however, is the strong inhibitory effect of *Wolbachia w*Mel strain on dengue establishment within mosquitoes. Lab and field examination of *Wolbachia* effects on the epidemiology of dengue revealed that the bacterial symbiont is able to approach fixation in mosquito populations within a few generations (Hoffmann et al. 2011). In addition to dengue, other human pathogens (e.g., Chikungunya, *Plasmodium*) are markedly reduced (1500-fold) in mosquitoes infected with *Wolbachia* compared to untreated individuals (Moreira et al. 2009; Walker et al. 2011), thereby providing an excellent example for how symbioses between insects and microbes could be harnessed as a means for the biological control of vector-borne diseases.

### Incompatible insect technique

Another potential *Wolbachia*-based approach to reduce both insect pests and vector-borne diseases is the incompatible insect technique (IIT). This procedure is analogous to the sterile insect technique (SIT) (Knipling 1955). Both methods rely on the mass release of sexually active but incompatible males into the wild that will mate with virgin females resulting in non-viable eggs (Laven 1967; Zabalou et al. 2004). SIT relies on various methods to achieve sterility in males, irradiation being the most common one. By contrast, IIT takes advantage of *Wolbachia*-induced cytoplasmic incompatibility (CI) or any other symbiont-induced reproductive incompatibility. *Wolbachia-*induced CI results in the death of embryos resulting from matings between *Wolbachia*-infected males and uninfected females (unidirectional CI) as well as from those involving individuals that are infected with different *Wolbachia* strains (bidirectional CI) (Werren 1997). In both examples, the release of infected males leads to a lower female fertility and can ultimately lead to the suppression of the population given enough time and constant release of incompatible males (Bourtzis et al. 2014).

The first successful application of IIT took place as early as in 1967 in Myanmar, where a population of the lymphatic filarial vector, the mosquito *Culex pipiens*, was eradicated, although the reasons behind the sterility were unknown at the time (Laven 1967). IIT has also been successfully tested against agricultural pests. Naturally, *Wolbachia*-free Mediterranean fruit flies (*Ceratitis capitata*) have been transinfected with a *Wolbachia* strain from the closely related cherry fruit fly *Rhagoletis cerasi* (Zabalou et al. 2004). This transinfection caused both unidirectional as well as bidirectional CI, opening the possibility for using it as an environmentally friendly pest control strategy. Equally promising are the results from Atyame and colleagues (2011). The *Wolbachia* strain wPip (ls) from *C*. *pipiens* was introgressed into *Culex quinquefasciatus* from four different islands of the South-Western Indian Ocean. In addition to 100 % embryo lethality from matings between sterile males and all tested field females, most crosses between introgressed females and field males were incompatible (Atyame et al. 2011).

However, the accidental release of *Wolbachia*-infected females could reduce the efficiency of the IIT by leading to the establishment of a viable *Wolbachia*-infected population as well as increase the risk of disease transmission since female insects are the transmitting vector. Therefore, different biological, genetic, and transgenic approaches to eliminate females early in the process by separating them from males have been developed (Laven 1967; Sweeny and Barr 1978; Condon et al. 2007; Brelsfoard et al. 2009). In addition to these, IIT itself could prevent the establishment of a transinfected population. The release of two reciprocally incompatible lines would result in most of the matings being incompatible (Bourtzis and Robinson 2006), thus reducing the number of potentially infectious females. An additional solution, and potentially the most promising, is to couple IIT with radiation-SIT (Bourtzis et al. 2014) since, for instance, tephritid flies as well as *Aedes polynesiensis* females can be sterilized with lower radiation than males (Bakri et al. 2005; Brelsfoard et al. 2009).

### Insect symbionts as probiotics

Several studies have shown that sterile mass-reared Mediterranean fruit flies subjected to SIT are less successful than wild males at competing for wild females (Juan-Blasco et al. 2013). In addition to this, irradiation of males for SIT also results in an altered gut microbiota as compared to non-irradiated males. Ben Ami and colleagues (2010) showed that supplementation of the diet with *Klebsiella oxytoca* as probiotics (one of the most abundant taxa in fruit flies Vienna 8 strain and in wild fruit flies) rescues male competitiveness by shortening their mating latency. Likewise, the addition of *Enterobacter* sp. to larval diet results in higher pupal and adult recovery as well as shorter developmental time in all life stages of male fruit flies (Augustinos et al. 2015). Therefore, further examination of insect symbionts as probiotics could be valuable in the efforts to develop more successful SIT applications.

Similarly, the use of probiotics can be a valuable tool in protecting honey bee (*Apismellifera*, Hymenoptera) populations, which are declining worldwide, probably due to a combination of pesticide use by humans and infection by parasites and pathogens (Cornman et al. 2012). For instance, the bacterium *Paenibacillus larvae* is responsible for the American foulbrood disease (AFB) within the insect’s gut, killing the larvae before pupation. Lactic acid bacteria (LAB) of the genera *Lactobacillus* and *Bifidobacterium* have recently been isolated from the honey stomach (Olofsson and Vásquez 2008) and are potential probiotic candidates for enhancing honey bee immunity. In vitro and in vivo studies indicate that LAB show total inhibition of *P*. *larvae* in agar diffusion assays and addition of LAB to the larval diet significantly reduced AFB infection (Forsgren et al. 2010). This effect may be mediated by a direct inhibition of pathogen proliferation or through stimulation of the host’s immune system (Evans and Lopez 2004). Other members of the honey bee gut community such as *Enterococcus* (Carina Audisio et al. 2011) and Actinomycetes (Promnuan et al. 2009) also produce antimicrobial compounds that have potential application in maintaining honey bee colony hygiene, as well as in preventing gut infection. Beneficial bacteria may thus provide interesting avenues for enhancing health and fitness of agriculturally important insects such as pollinators.

### Symbiont-produced compounds with antimicrobial activity

Antimicrobial secondary metabolites find important applications in human medicine and agriculture. However, the increasing resistance of human pathogens and the reduced discovery rate of novel compounds pose significant problems that threaten to result in the reappearance of human diseases that were thought to be defeated. In this context, insect-associated microbes present promising sources of novel bioactive compounds that are only beginning to be discovered and exploited (Dettner 2011). In particular, defensive insect-bacteria symbioses are interesting targets for natural products discovery, as the involved secondary metabolites have been tested over millions of years by natural selection for their efficacy against antagonists as well as for the lack of harmful side effects on the eukaryotic host (Flórez et al. 2015). In general, symbiont-produced defensive compounds are employed by insects in two different contexts: (i) as a protection of the host or its offspring against antagonistic micro- or macroorganisms or (ii) as weed killers in insect fungiculture (Kaltenpoth 2009; Ramadhar et al. 2014).

Microbial symbionts providing chemical defense to the host against predators, parasites, parasitoids, and pathogens occur in several insect taxa, including beetles, psyllids, planthoppers, and solitary wasps. In staphylinid beetles of the genus *Paederus*, symbiotic *Pseudomonas* bacteria produce the polyketide pederin that deters predatory wolf spiders (Kellner 2001; Kellner 2002; Piel 2002). A similar compound called diaphorin has recently been found to be produced by an intracellular symbiont of psyllids and suspected to confer protection against as yet unknown predators (Nakabachi et al. 2013). In another hemipteran insect, the brown planthopper *Nilaparvata lugens* (Delphacidae), an *Enterobacter* symbiont produces the antimicrobial compound andrimid with activity against pathogens of the planthopper’s host plant (Fredenhagen et al. 1987). Finally, two different insect taxa—a group of solitary wasps and a weevil—employ symbiont-produced antimicrobials for protection of their developing offspring against mold fungi. The “*Candidatus* Streptomyces philanthi” symbionts of solitary beewolf wasps in the genera *Philanthus*, *Trachypus*, and *Philanthinus* produce a mixture of streptochlorin and at least eight different piericidins that defend the larva inside the cocoon against opportunistic mold fungi (Kaltenpoth et al. 2005; Kroiss et al. 2010; Kaltenpoth et al. 2014). And the leaf-rolling weevil *Euops chinensis* teams up with the fungus *Penicillium herquei* that produces (+)-scleroderolide and thereby protects the larval cradle against microbial antagonists (Wang et al. 2015).

Analogous to human agriculture, the domestication of fungal cultivars for food has evolved independently in several insect lineages. Due to the necessity for protecting the fungus monoculture from pathogens, these systems are particularly promising potential sources for bioactive metabolites produced by the insect themselves or associated symbionts (Ramadhar et al. 2014). Concordantly, actinobacterial symbionts of different fungus-growing ant species have been found to produce a range of secondary metabolites with general antimicrobial activity or targeting specific fungal antagonists. These compounds include dentigerumycin (Oh et al. 2009a), pseudonocardones A–C, 6-deoxy-8-O-methylrabelomycin, and X-14881 E (Carr et al. 2012a), nystatin P1 (Barke et al. 2010), candicidin (Haeder et al. 2009), as well as actinomycins, antimycins, and valinomycins (Schoenian et al. 2011). Furthermore, a *Streptomyces* strain associated with the fungus-growing bark beetle *Dendroctonus frontalis* produces the antifungal compound mycangimycin (Oh et al. 2009b). Finally, microtermolides A and B were isolated from a termite-associated *Streptomyces*, but showed no bioactivity against the tested bacterial and fungal strains (Carr et al. 2012b). Although to our knowledge, none of the compounds involved in these symbiotic associations has so far been exploited for clinical application, several substances show interesting antimicrobial, antiparasitic, or anti-cancer activities and may therefore be of interest for human medicine. Future studies on insects with life histories that entail particularly high exposure to pathogens (e.g., fungicultural systems, insects that mass-provision their offspring and/or develop within the soil) will likely uncover additional defensive symbioses and bioactive natural products of potential value for human application.

### Insect symbionts as a source of digestive enzymes

Insects show remarkable adaptations to the exploitation of diverse nutritional resources, owing to the wide diversity of digestive enzymes produced by the insects themselves as well as the metabolic capabilities of symbiotic microorganisms that overcome the host’s nutritional limitations. In addition to the supplementation of essential nutrients such as amino acids, vitamins, and sterols, gut symbionts can also provide beneficial digestive enzymes when the host’s diet is specialized, contains refractory substrates, and is deficient in nutrients and/or when insects colonize novel niches for which their own metabolic repertoire is inadequate. The following sections deal with biotechnologically relevant digestive enzymes produced by microbial symbionts in insects.

#### Cellulases, ligninases, and pectinases

Cellulases (cellulose-hydrolyzing enzymes, including endoglucanases, exoglucanases, and β-glucosidases) are a major group of industrial enzymes with applications in textile processing, paper recycling, and detergent production and in the food industry. Biotechnologically important cellulose-producing fungi and bacteria include the free-living taxa *Trichoderma reesei*, *Humicola insolens*, *Aspergillus niger*, *Bacillus subtilis*, *and Clostridium* spp. (Bhat 2000; Phitsuwan et al. 2013). However, there are numerous reports on insects hosting symbiotic microorganisms to digest plant fibers, which may yield novel cellulases as well as other biotechnologically important proteins such as carbohydrate binding modules (CBMs) and enzymes of the family AA9 (formerly GH61) that synergistically enhance the efficacy of existing cellulolytic enzymes and have recently been discovered from insect symbionts (Takasuka et al. 2013; Poulsen et al. 2014). Particularly, termites host an extensively studied and biotechnologically promising gut community of cellulase producers, which can digest up to 74–99 % of the ingested cellulose and about 65–87 % of the hemicellulose (Breznak and Brune 1994). In addition to the termites’ endogenous cellulases (Watanabe and Tokuda 2010), lower termites host cellulolytic protists, while higher termites harbor hindgut bacteria (Spirochetes, Bacteroidetes, Firmicutes, and/or Fibrobacteres) to degrade lignocellulose, with the host enzymes acting on the amorphous regions of cellulose and the symbiotic enzymes targeting the crystalline regions (Brune 2014).

Lignocellulose digestion requires diverse glycoside hydrolases (GH) to break down the different components of plant cell wall before cellulases can come into play. Current industrial processes use crude extracts of cellulolytic fungi (such as *T*. *reesei*) not only for economic reasons, but also due to the synergism of the multiple enzymes they contain (Fischer et al. 2013). Importantly, termite guts also contain a large diversity of GH enzymes (Poulsen et al. 2014); *Nasutitermes* sp. gut symbionts contribute 125 GH5 cellulases, 101 GH10 xylanases, and several GH8, GH9, and GH45 endoglucanases (Shi et al. 2013). Symbionts in *Nasutitermes takasagoensis* use the cellulosome (an extracellular multiprotein complex with a single cellulose binding domain) to digest cellulose (Tokuda et al. 2005), similar to the biotechnologically important *Clostridium thermocellum*, one of the most efficient cellulolytic bacteria. Similarly, a *Streptomyces* sp. associated with wood wasps secretes multiple enzymes including endo- and exoglucanases, with a high biomass-degrading activity comparable to that of *T*. *reesei* (Takasuka et al. 2013). Recently, cellulases have also been discovered from symbionts associated with pine beetles (Book et al. 2014), sugarcane weevils (Rinke et al. 2011), and a range of other insects, including Orthoptera, Blattaria, Hemiptera, Coleoptera, Hymenoptera, Lepidoptera, and Diptera (reviewed in Calderón-Cortés et al. 2012).

Ligninases are also important enzymes in processing lignocellulose. While animals generally lack these enzymes, several fungi and bacteria are able to degrade lignin, producing veratraldehyde in this process, a flavorant and odorant with a pleasant woody fragrance. Several wood-feeding insects (Asian long-horned beetles, Pacific dampwood termite, fungus-growing termites) host symbiotic soft-rot fungi in their guts (Geib et al. 2008), either to enable efficient utilization of cellulose or to degrade lignin itself as a source of nutrition (Hyodo et al. 2003), both important biotechnological applications.

Similarly, pectinase, an enzyme that breaks down pectin (a heteropolysaccharide found in plant cell walls), has biotechnological potential in extracting fruit juice (e.g., apple juice) and in wine production. Honey bees host symbiotic Gamma-Proteobacteria that possess the genetic potential for pectin degradation and show in vitro production of pectinase to break down pollen cell walls (Engel and Moran 2012). In leaf-cutting ants, pectinolytic enzymes that are ingested from the fungal cultivar pass unaffected through the ant gut and are finally applied to the plant substrates used for fungal cultivation (Schiøtt et al. 2010).

#### Other digestive and nutritionally important enzymes

Lipases and proteases have important applications in the production of biodiesel (conversion of vegetable oil to alcohol esters), synthetic polymers, pharmaceuticals, agrochemicals, cosmetics, flavoring compounds, as well as in bioremediation such as decontamination of wastewater and oil polluted soils. Currently, *Candida* spp., *Pseudomonas* spp., and *Rhizopus* spp. are important sources of lipase production, but several other microbes are used as well (Pandey et al. 1999). Symbiotic microorganisms with potential lipase and/or protease activity have been isolated from insects, including *Bacillus*, *Staphylococcus*, *Pseudomonas*, *Fusarium solani*, *Candida fermentati*, *Yarrowia lipolytica*, and yeast-like symbionts from silkworm, long-horned beetles, cigarette beetles, cottony maple scale insects, and burying beetles, respectively (Brues and Glaser 1921; Vega and Dowd 2005; Park et al. 2007; Feng et al. 2011; Scully et al. 2012; Kaltenpoth and Steiger 2014). Unfortunately, however, information on their relevance for host fitness or their potential for biotechnological application remains limited. Extracellular lipase-producing symbionts are especially interesting from a biotechnological perspective, such as lipases secreted by yeast-like symbionts associated with deathwatch beetles, wood-boring beetles, and fruit flies, which play a role in the hosts’ nutrition (Gonzalez 2014). Gut bacteria in the soybean-feeding velvetbean caterpillar *Anticarsia gemmatalis* produce proteases (“general” and serine proteases) that could block protease inhibitors present in soybean leaves (Visôtto et al. 2009). Basidiomycete fungi associated with ants also secrete proteases that help detoxification and nutrient assimilation (Gonzalez 2014).

In addition to the production of biotechnologically interesting digestive enzymes, insect symbionts have been implicated in the biosynthesis of nutritional compounds of applied value, particularly B vitamins, e.g., in lice, tsetse flies, sharpshooters, and aphids (reviewed in Douglas 2009). However, the inability to isolate and culture these symbionts on artificial media severely limits their biotechnological potential. By contrast, cultivable (and therefore biotechnologically more interesting) B vitamin-producing actinobacterial symbionts are known to occur in firebugs and African cotton stainers (Salem et al. 2014), and culturable yeast-like symbionts provide anobiid beetles and wood wasps with sterols, vitamins, and/or essential amino acids (Pant and Fraenkel 1954; Bismanis 1976).

### Potential role of insect symbionts in bioremediation and detoxification

Bioremediation is the elimination, attenuation, or transformation of polluting or contaminating substances by the use of biological processes. In addition to the applied potential, understanding how organisms metabolize such compounds has important implications for understanding ecological adaptation and the evolution of resistance against pesticides.

Insects are exposed to toxic natural products (especially plant secondary metabolites) as well as noxious compounds from human activities (pollutants and insecticides). Detoxifying enzymes providing resistance against both groups of chemicals can be produced by the hosts themselves or by microbial symbionts (Douglas 2013). Evidence for fungal symbionts involved in detoxification across several insect orders was reviewed by Dowd (1992); symbiotic fungi belonging to the genera *Candida*, *Aspergillus*, *Amylostereum*, *Phanerochaete*, and *Xylaria* are present in bark beetles, wood wasps, leaf-cutting ants, and various other insects, where they may aid in the degradation of terpenes, tannins, toxic esters, phenolics, chlorinated hydrocarbons, alkaloids, and quinones. For example, mycetomes present between the foregut and midgut of the cigarette beetle *Lasioderma serricorne* contain symbiotic yeasts that produce hydrolytic enzymes acting on toxic phenolic compounds such as tannins, as well as on alkaloid esters (Dowd and Shen 1990). The symbionts are thought to be closely related to plant pathogens that evolved similar mechanisms to overcome plant toxins. Similarly, fenitrothion-degrading bacteria (Burkholderiales) have been isolated from the gut of bean bugs (*Riptortus pedestris*) and stinkbugs (*Leptocorisa chinensis*) and shown to confer insecticide resistance to their hosts (Kikuchi et al. 2012). The mountain pine beetle *Dendroctonus ponderosae*, a herbivore of conifers, hosts a bacterial gut community dominated by *Pseudomonas*, *Rahnella*, *and Burkholderia* that display terpene-degrading genes (Adams et al. 2013), which putatively benefit the host in detoxification of conifer defenses. An important bioremediation case study comes from bacterial strains of *Enterobacter* and *Bacillus* that were isolated from the gut of the waxworm *Plodia interpunctella* that naturally feed on beeswax. Within 60 days, these bacteria were capable of degrading 6.1 to 10.7 % of polyethylene—a substrate with long-chain hydrocarbons as in their natural diet—when fed as the sole carbon source to the larvae (Yang et al. 2014). The breakdown products were reportedly water soluble, but were not further characterized in the study.

Two detoxifying enzymes with particular application potential in biotechnology are linamarase (β-d-glucosidase) and laccase. Linamarase produced by the bacterial gut community (*Acinetobacter* sp., *Bacillus* sp., *Klebsiella* sp., *Alcaligenes* sp.) of variegated grasshoppers (*Zonocerus variegatus*) acts on the cyanogenic glucosides of their food plants (Idowu et al. 2009). This enzyme can be used for the reduction of free cyanide levels in the fermentation of cassava fruits that contain cyanogenic glycosides (Ikediobi and Onyike 1982). Laccases, enzymes that catalyze the oxidation of aromatic compounds (phenols and amino-phenols), are widely used in organic synthesis, bioremediation, textile industry, wine stabilization, and biosensors for immunoassays (Kunamneni et al. 2008). In a symbiotic context, these enzymes are produced by fungal symbionts of the leaf-cutting ant *Acromyrmex echinator* (De Fine Licht et al. 2013). Interestingly, the symbiont-produced enzyme is consumed by the ants and remains active during the passage through the gut, where it is used to detoxify phenolic compounds in the plant material that are then supplied to the fungus for nutrition (De Fine Licht et al. 2013). *Actinobacteria* isolated from the termite *Amitermes hastatus* gut also showed high laccase activity (Le Roes-Hill et al. 2011), highlighting the potential of insect symbionts as producers of biotechnologically relevant detoxifying enzymes.

## Conclusions

Insect symbionts constitute a rich and mostly untapped source of bioactive small molecules as well as digestive enzymes of potential biotechnological value. Even though their exploitation is currently hampered by the unculturability of most symbionts, advances in culturing techniques as well as genomic and genetic tools for the identification and heterologous expression of genes of interest may overcome this hurdle. Furthermore, a large diversity of facultative associates is experimentally and/or genetically tractable and could be of more immediate applied value. In addition to exploiting their metabolic capabilities, insect symbionts can be used to promote insect health as well as to target and control agricultural pest insects and vectors of medically important human diseases in an environmentally friendly way. Overall, increasing research efforts in the areas of insect ecology and symbiosis not only promise to uncover interesting new symbiotic alliances but may also prove valuable in the continued effort to find new sources of biotechnologically important molecules and enzymes. Specifically, targeted searches for compounds with particular applied value (e.g., antibiotics, detoxifying enzymes, cellulases, lipases, etc.) may benefit from being guided by the knowledge on host-symbiont ecology, which has the potential to predict particularly promising systems for exploration.
